# Radioactive Iodine-125 in Tumor Therapy: Advances and Future Directions

**DOI:** 10.3389/fonc.2021.717180

**Published:** 2021-09-30

**Authors:** Shuhua Wei, Chunxiao Li, Mengyuan Li, Yan Xiong, Yuliang Jiang, Haitao Sun, Bin Qiu, Christopher J. Lin, Junjie Wang

**Affiliations:** ^1^ Department of Radiation Oncology, Peking University 3rd Hospital, Beijing, China; ^2^ University of Southern California, Los Angeles, CA, United States

**Keywords:** brachytherapy, iodine-125, low-dose-rate, high-dose-rate, 3D printing template, tumor

## Abstract

Radioactive iodine-125 (I-125) is the most widely used radioactive sealed source for interstitial permanent brachytherapy (BT). BT has the exceptional ability to deliver extremely high doses that external beam radiotherapy (EBRT) could never achieve within treated lesions, with the added benefit that doses drop off rapidly outside the target lesion by minimizing the exposure of uninvolved surrounding normal tissue. Spurred by multiple biological and technological advances, BT application has experienced substantial alteration over the past few decades. The procedure of I-125 radioactive seed implantation evolved from ultrasound guidance to computed tomography guidance. Compellingly, the creative introduction of 3D-printed individual templates, BT treatment planning systems, and artificial intelligence navigator systems remarkably increased the accuracy of I-125 BT and individualized I-125 ablative radiotherapy. Of note, utilizing I-125 to treat carcinoma in hollow cavity organs was enabled by the utility of self-expandable metal stents (SEMSs). Initially, I-125 BT was only used in the treatment of rare tumors. However, an increasing number of clinical trials upheld the efficacy and safety of I-125 BT in almost all tumors. Therefore, this study aims to summarize the recent advances of I-125 BT in cancer therapy, which cover experimental research to clinical investigations, including the development of novel techniques. This review also raises unanswered questions that may prompt future clinical trials and experimental work.

## Introduction

As one of the main techniques for the delivery of radiotherapy, brachytherapy (BT) is the implantation of radioactive sources into patients’ bodies through intraluminal or interstitial applicators. BT can deliver extremely high prescribed doses inside the target lesion with minimal dose to the adjacent normal tissues, which are impossible to achieve with external beam radiotherapy (EBRT) or stereotactic body radiation therapy (SBRT). Because of this, BT has been deemed as the most conformal of therapy techniques. BT has recently been highly successful in treating cerebral, oral/maxillofacial, pulmonary, hepatic, pancreatic, and, most commonly, prostate cancer. Increasing number of studies have investigated and confirmed the possibility and safety of computed tomography (CT)-guided radioactive iodine-125 (I-125) BT in head and neck, thoracic, abdominal, pelvic, spinal primary, and even metastatic cancer in the last two decades (1–5).

In general, BT is performed by permanent seed implants [also as low-dose rate (LDR) or high-dose rate (HDR)] after loading techniques (6). Both have served as auxiliary BT boosts to complement EBRT for better local control (LC) (7). While other compounds such as Cs-131, Cs-137, Pd-103, and Ir-192 are all common choices for BT, LDR-BT is typically performed with I-125 (28.4 keV of average energy, 59.4 days of half-life).

The endeavor to refine the procedure of implanting or placing I-125 to the target lesion has lasted several decades. Both Ultrasonography (US) and CT facilitated the utilization of I-125 BT in clinical practice (8, 9). The combination of I-125 BT with CT simulators enabled real-time dosimetry optimization during operations. Additionally, some scholars innovatively introduced three-dimensional printing templates (3D-PTs) into the seed implantation process to reduce arrangement errors during the insertion of needles that greatly increased precision and efficiency (2). Furthermore, artificial intelligence (AI) navigation and absorbable stranded seeds have played a recent role in the development of I-125 BT to improve reproducibility and prevent the migration of seeds as the result of tumor regression (10). With the development of I-125 seed-loaded stents, I-125 was able to be reliably and feasibly used for the first time in the treatment of esophageal, biliary, and bronchial cancer obstructions (11–15). All these refinements have enhanced the accuracy, feasibility, and applicability of I-125 BT.

I-125 BT has long been used as a radical modality for early-stage prostate cancer and used in inoperable brain tumors and head and neck cancer (7, 16). Some advantages of interest to translate and use in other tumors have included image guidance, minimal invasion, local dose escalation, and organ preservation. Moreover, the expert consensus and standardized procedure of 3D-PT-assisted I-125 BT on pancreatic cancer and lung cancer have popularized this interventional strategy (17, 18). Other usages of I-125 BT have included salvage for recurrent rectal cancer after multiple lines of treatment as recommended by National Comprehensive Cancer Network (NCCN) guidelines (19, 20) and the use of I-125 seed stents successfully in esophageal cancer that have presented fresh opportunities for mitigating cancer-related symptoms (11). In these cases, I-125 BT has turned out to be highly effective. However, although a variety of clinical trials have evaluated the efficacy, safety, and side effects of I-125 in a variety of carcinomas, there are still numerous problems remaining for these tumors. For instance, randomized clinical trials (RCTs) are still needed to compare I-125 BT with additional therapies regarding long-term clinical efficacy. Results of the biological effects of I-125 have long been discouraging. Additionally, the role of locally continuous LDR-BT by I-125 in immunomodulation is largely unknown. Encouragingly, the coordinated immune response triggered by local EBRT might help illuminate research on the immunomodulation of locally continuous LDR-BT by I-125 (21). Thus, the purpose of this review is tantamount to sum up current research of interest for I-125 BT, which involves the discussion of technique, preclinical studies, translational medicine, and clinical work, hopefully informing future investigations.

## The Refinement in the Procedure of I-125 Brachytherapy

The intent of I-125 BT, in general, is to increase deliverable radiation doses to the target lesion while reducing the exposure of uninvolved normal tissues, as well as to reduce the side effects attributable to I-125. As the progressive development of technology facilitates clinical practice, the application of the I-125 seed in various clinical scenarios is becoming increasingly safe and reliable. As a minimally invasive interventional modality, the security and accuracy of I-125 BT procedures must be taken into consideration, as well as the stability and expected dose distribution of radioactive sources inside the target. Thus, we summarized the progress related to I-125 BT and presented some new ideas.

## Advances in Imaging Techniques

Initially, clinicians surgically placed radioactive sources inside the lesion for some unresectable cancers (22). Later on, in order to meet the need for minimal invasion, US-guided I-125 BT was successfully applied in pancreatic cancer (8). However, for other tumors, the low resolution of US imaging created several challenges for clinicians performing I-125 BT, which included the lack of 3D imaging, template assistance, freehand operation, an uncontrollable arrangement of needles, and unachievable dosimetry optimization. To overcome these problems, radiation oncologists attempted CT-guided I-125 BT, which had three advantages, including enhanced visualization of the target, 3D digital image reconstruction, and real-time monitoring of needle arrangements. Under some circumstances, magnetic resonance imaging (MRI) and ^18^F-fluorodeoxyglucose positron emission tomography (FDG-PET) data were required for integration with CT images to precisely define the margin of tumor to avoid any diagnostic misunderstanding of the tumor with adjacent edema and atelectasis (23, 24). Based on CT images, the preoperative plan and intraoperative and postoperative dose verification guaranteed the accuracy of the entire process. However, as clinical popularization of CT-guided I-125 BT increases, several variables including organ movement, interference of organs at risk (OARs), unexpected extra exposure of radiation on patients, the long training period of a qualified operator, and the establishment of standardized procedure should be considered.

## The Incorporation of Three-Dimensional Printing Template With CT Guidance

**Figure 1:**
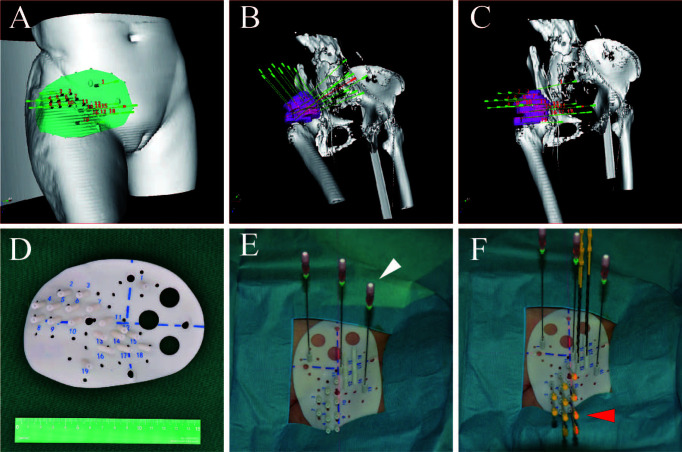
The design of 3D-PNCT based on CT-simulation for a patient with recurrent ovarian cancer. **(A)** The 3D digital model of 3D-PNCT. **(B, C)** Design the optimal puncture paths and needles angle on 3D digital model. **(D)** The real product of 3D-PNCT contains cooridinate axis for alignment, holes for stable needle and puncture needle. **(E, F)** The immoblized 3D-PNCT with stable needles (white arrow) and catheters (red arrow) before 125I seeds implantation. 3D-PNCT, three-dimensional printing non-coplanar template.

**Figure 2:**
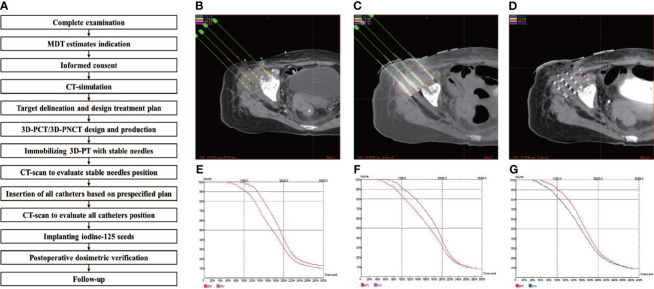
Example of the successive steps in 3D-PT assisted I-125 BT procedure. **(A)** the implantation work-flow. CT-image acquisition for target delineation, treatment planning and optimization: **(B)** preoperation, **(C)** intraoperation, and **(D)** postoperation; DVH for treatment planning optimization and verification: **(E)** preoperation, **(F)** intraoperation, and **(G)** postoperation.

Although radiation oncologists carefully design the puncture path, needle angles, and I-125 seed distribution inside the targets on the preoperative plan, the actual puncture is always unsatisfactory under CT guidance due to the complicated anatomical structure of specific sites, organ movement, and OAR interference. In some cases, in order to reduce the risk of puncture-related hemorrhage, nerve damage, and pneumothorax, the pre-plan-designed puncture path must be extremely elaborate, which makes the needle insertion difficult to duplicate and is a huge challenge for operators. Fiducial markers, analogous to the surface tattoo for EBRT, can aid this process by linking the pretreatment plan with actual real-time operation (25, 26). In 2012, Huang et al. (27) innovatively introduced 3D-PT into I-125 BT in the treatment of head and neck carcinoma and then extended it to thoracic, abdominal, and pelvic cancer in 2015. Three-dimensional printing template is generally classified into two types: three-dimensional printing coplanar template (3D-PCT) and three-dimensional printing non-coplanar template (3D-PNCT). Three-dimensional printing coplanar template is suitable for situations where all needle tracts are kept in parallel, while 3D-PNCT is favored in more complex situations where multidirectional punctures are required to optimize dose distribution for irregular tumors (28, 29). According to the preoperative plan and CT imaging, a 3D-digital individualized template can be established that details the superficial anatomic characteristics; positioning marks; puncture sites; needle orientation; and X, Y coordinate information (**Figures 1A–C**). Then, taking into account this 3D-digital template, physical templates can be manufactured by 3D rapid prototyping equipment utilizing photo-curable resins (**Figures 1D–F**). Notably, a real-time intraoperative CT scan was performed to monitor the needle’s position, and the postoperative scan was immediately conducted after seed implantation (**Figure 2**). There is no edema within the tumor in such a short time. Therefore, there is no need to consider the impact of edema. Thus, the incorporation of 3D-PT into CT-guided I-125 BT helped ensure more accurate needle arrangement and achieved more conformal radiation delivery, which led to the conception of stereotactic ablative BT (SABT).

## Stranded Seeds Reduce Seed Migration

Despite more accurate needle arrangements using methods such as SABT, the movement of prelocalized seeds from their predesigned seats is a problem in the implementation of loose seeds. This movement, called seed migration, is often a function of how I-125 gradually alters the tumor volume and tumor microenvironment through necrosis and apoptosis of the tumor site. Unexpected seed migration, which results in worse tumor dosimetry distribution, may reduce the LC rate and increase the risk of recurrence or metastasis. Therefore, seed migration leads to worse clinical outcomes and complications such as lung or cardiac seed embolization (30). Additionally, organ movement and operating errors can lead to seed displacement. Seed loss, seed migration, and seed displacement remain unsolvable with the use of loose seeds for radiation oncologists. An option to overcome seed migration is to link the seeds. Several clinical studies, which created novel intraoperative built custom-linked seeds for the treatment of prostate cancer, were able to demonstrate a lower risk of seed migration, more stable dose distribution, and increased no biochemical evidence of disease in stranded seeds compared to loose seeds, with slightly longer operation time in the stranded-seeds group (10, 31, 32). However, the optimal material to link seeds has yet to be well defined.

## I-125-Loaded Stent

It has long been difficult to perform I-125 BT in hollow organs, such as the esophagus, trachea, and bile duct until the emergence of I-125-loaded SEMS, which is a favorable option to relieve cancer-related symptoms that always deteriorate the patient’s quality of life, such as refractory dysphagia, dyspnea, and jaundice (11, 14). Although stents can mitigate these symptoms to some extent through physical expansion, the high risk of recurrence and restenosis and unsatisfactory overall survival (OS) are issues still difficult to resolve. Therefore, by integrating I-125 onto the mesh of SEMS, solving these issues was made possible.

However, to implement I-125 safely and effectively in SEMS, the number of seeds must be carefully determined by the dose required. In practice, radiation oncologists determine the prescribed dose and total quantity of I-125 through a pretreatment plan, assemble I-125 into a sheath, and load it onto the stent before the intervention therapy. Under the guidance of fluoroscopy and US, the I-125-loaded stent is placed into the target lesion through a natural cavity or percutaneous puncture. Of note, several clinical studies have assessed the efficacy of I-125-loaded stent on tumors with refractory symptoms, suggesting that I-125-loaded stent can significantly mitigate cancer-related symptoms, prolong survival, reduce the risk of restenosis, and improve quality of life (11, 12).

## I-125 Brachytherapy *vs.* High-Dose Rate

The radiobiological mechanisms of BT have been poorly understood with most available data focusing on dose rate effect (33). As a form of continuous LDR-BT, I-125 BT dose rate is dependent upon radioactivity of the I-125 seed and, at 1 Gy/h dose rate, is approximately isoeffective with fractionated radiotherapy using 2-Gy fractions (34). Human tumor cell lines show a wide range of radiosensitivities to LDR-BT of 1 Gy/h. It may be produced by DNA damage due to clusters of ionization events or perhaps by damage to hypersensitive parts of the genome (34). Radiosensitivity of human tumor cells to both LDR and HDR irradiation is genotype dependent (35). Low-dose hyper-radiosensitivity or increased radioresistance was observed in rat colon progressive cells; the former may be involved with impairments in non-homologous end-joining repair and was independent of DNA-dependent protein kinase (DNA-PK), while the presence of increased radioresistance was coincident with the presence of PRKDC protein and functional DNA-PK activity (36, 37).

Theoretically, tumor cell repopulation or proliferation may occur during the long period taken to deliver the full radiation dose in LDR-BT. The balance between tumor DNA damages and the ability to repair sublethal damages was lethal for controlling tumor proliferation (38). The late-reacting normal tissues are believed to have lower a/b ratios than tumor and, therefore, will be spared when dose rate decreases, though tumor DNA damages can also be repaired to some extent (39). LDR-BT can be expected to give a better therapeutic ratio, but this generalization will only hold if tumor repopulation is small during the course of the treatment (38). For dose rates ranging from 0.3 to 1 Gy/h, the possibility of repopulation during treatment is negligible (38). However, I-125 seed BT at an LDR of 2.77 cGy/h also showed more effective induction of cell apoptosis and G2/M cell cycle arrest in colorectal cancer cells compared with 6 MV X-ray at an HDR of 4 Gy/min (40). A similar result was shown in pancreatic cancer cells (41). Using typically prescribed doses of I-125 BT, with a relative biological effectiveness (RBE) of 1.4 at dose rates of about 0.07 Gy/h, appears to be better for treating radiosensitive tumors with long doubling times and that shrink rapidly (42, 43). Therefore, the optimum use of I-125 BT may rely on selecting relatively radiosensitive tumors arising in anatomical sites where late reactions are dose-limiting (44). Furthermore, shorter-lived radionuclides are more versatile for achieving reasonable clinical results for a wide range of tumor types, the theoretically derived optimum half-lives typically range from around 0–5 days for fast-repopulating tumors to approximately 14–50 days for slow-growing tumors (45). Notably, given the increasing reports of HDR irradiation equivalent to LDR treatments, HDR-BT (usually with dose rate above 12 Gy/h) may have an advantage over LDR-BT for rapidly growing tumors with increasing capacity for cellular repair (46–48).

Clinically, as the main approaches in the field of modern BT, LDR-BT and HDR-BT own benefits over each other on non-overlapping aspects (**Table 1**). In terms of medical providers, although a shielded room is not required for LDR-BT compared with HDR-BT, radiation exposure of clinicians during the LDR-BT operation remains a concern. The operation room for LDR-BT could be integrated with the CT simulation room, while extra initial capital equipment for HDR-BT must be considered. Moreover, radioactive sources for LDR-BT were customized for each patient, while the same source for HDT-BT could be used for different diseases in 3–4 months. Additionally, an excellent LDR-BT operation depends on the experience and skill of operators to a much more extent than HDR-BT. Thus, the individual 3D-PT was introduced into the procedure of LDR-BT to minimize the dependence on the operator. For patients, in contrast to HDR-BT, the shorter treatment period and low cost of LDR-BT seem more favorable for patients who live far from a cancer center or lack basic medical insurance. Several retrospective and prospective studies suggested the comparable outcome among HDR-BT and LDR-BT, while HDR-BT seems to have fewer acute irritative symptoms. Given that most published studies comparing the efficacy of LDR-BT and HDR-BT focused on prostate cancer, prospective studies comparing these two modalities in various diseases will be necessary for the future.

**Table 1:** Overview of the applications of mesenchymal stromal cell-derived extracellular vesicles (MSC-EVs) in clinical studies.

	LDR-BT	HDR-BT
For physician and hospital		
Dose coverage	Superior conformality, like HDR-BT	Superior conformality, like HDR-BT
Requirement for Shielded room	No	Yes
Utility of radioactive source	Customized	Reusable
Capital expenditure	Low	High
Dependence of operator	High	Low
Treatment planning	Postoperative dosimetry evaluation is required	Without postoperative dosimetry evaluation
Radiation exposure to staff	Yes, could be protective	No
Evidence	Excellent	Inferior to LDR-BT
Uniform consensus dose	Yes, such as 140-180Gy for I-125	No, multiple fractionation options
Treatment modality	1. Monotherapy for low-risk disease 2. Combined with surgery, EBRT or other treatment for intermediate-high risk disease. 3. Salvage for recurrent or relapse cancer after multiple treatment. 4. Combined with self-expandable stent.	1. Monotherapy for low-risk disease, like LDR-BT. 2. Combined with surgery, EBRT or other treatment for intermediate-high risk disease, like LDR-BT. 3. Salvage for some diseases.
Template assistance	3D-printing coplanar/non-coplanar template	Conventional template
Seed migration	Yes, but rare.	No
For patients		
Cost for treatment	Lower than EBRT	Lower than EBRT
The placement of source	Permanent	Temporary
Procedure related event	Rare	Rare
Treatment period	Singe implant in 2h in a single day	Several implant in 2h in 1-5 days
Toxicity	1. Overall incidence similar to HDR-BT.	1. Overall incidence similar to LDR-BT.
	2. Longer duration (2–4 months) than HDR-BT.	2. Shorter duration (4–6 weeks) than LDR−BT.
	3. Grade 3-4 toxicities are rare.	3. Grade 3-4 toxicities are rare.
Indications	Including almost all solid tumor such as brain tumor, eye tumor, lung cancer, head and neck cancer, recurrence located in operation restricted area and even malignant obstruction, besides tumor in hollow organs.	Narrower than LDR-BT, including prostate cancer, gynecological cancer, rectal cancer, sarcoma, breast cancer, non-melanoma skin cancer, penile cancer etc.

LDR-BT, low-dose rate brachytherapy; HDR-BT, high-dose rate brachytherapy; EBRT, external beam radiotherapy; CT, computed tomography.

## Physical Aspects

Accurate dose delivery is the guarantee of the curative effect of radiotherapy. American Brachytherapy Society (ABS) and American Association of Physicists in Medicine (AAPM) reported (TG-43) the recommended dosimetry values, including D90 >100%, V100 >90%–95%, and V150 <50%–60%. As a result, many studies have compared dosimetry differences between different BT modalities to investigate the accuracy of I-125 BT. For instance, Li et al. (49) reported that intraoperative planning was superior to the preplanning technique, *via* comparing the difference of dosimetry parameter between preplanning and intraoperative planning in I-125 BT for lung cancer. They suggested that V100, V150, and V200 for the intraoperative planning technique were significantly higher than that for the preplanning technique (95.65% *vs.* 88.86%, 76.47% *vs.* 69.23%, and 59.80% *vs.* 28.30%, respectively, p < 0.01). In addition, intraoperative planning had a significantly higher coverage index, conformity index plan, and quality index (p < 0.05) and a lower dose homogeneity index compared with the preplanning technique (49). When it comes to the improved accuracy brought by 3D-PT, Liu et al. (50) indicated the comparable dosimetry parameters between preoperative plans with postoperative plans in 3D-PT-guided I-125 BT for recurrent high-grade gliomas, regarding D90 (152.1Gy *vs.* 151.7Gy, p > 0.05), V100 (96.8% *vs.* 97.0%, p > 0.05), and V200 (49.1% *vs.* 48.9%, p > 0.05). When comparing I-125 LDR-BT with HDR-BT in prostate cancer, a randomized trial suggested that D90, V100, and V150 were significantly higher in the LDR-BT group than those in the HDR-BT group (122% *vs.* 110%, p < 0.05; 99% *vs.* 98%, p < 0.05; 61% *vs.* 32%, p < 0.05). Moreover, in this study, D10 and D30 for urethra and D10 for rectum were significantly higher in LDR-BT group than HDR-BT group (133% *vs.* 114%, p < 0.05; 128% *vs.* 111%, p < 0.05; 88% *vs.* 67%, p < 0.05) (51). Thus, although both I-125 LDR-BT and HDR-BT are able to provide a favorable dose coverage, a slightly higher dose to the urethra and rectum was seen in I-125 BT, which might account for the higher incidence of genitourinary (GU) and gastrointestinal (GI) toxicity in LDR-BT. Furthermore, a retrospective analysis (25 patients with pancreatic cancer) indicated that 3D-PCT-assisted I-125 BT had a dosimetry advantage in V100 (91.05% ± 4.06% *vs.* 72.91% ± 13.78%, p < 0.05) compared with implantation by freehand. That is to say, the current BT, assisted by 3D-PT and guided by CT scan, has achieved a perfect match between postoperative dose verification with preoperative planning. No significant differences were observed between preoperative planning design and postoperative dose verification regarding several dosimetry indexes. The accuracy was improved more than 90%. The introduction of 3D individualized templates is beneficial to realize individualize treatment and do away with the dependence on operator experience (**Figure 2**).

Notably, compared with HDR-BT, one of the disadvantages of I-125 BT is the exposure of clinicians during the operation and subsequently exposure of patients’ relatives and population. Licciardello et al. (52) conducted Ќ measurements [the air Kerma rate Ќ (mGy/h), which is regularly measured and recorded for every patient from the day after the implant to the day before hospital discharge] at various distances from the patient surface to analyze the exposure rate in the proximity of patients and compute the effective doses to relatives and population, which was used to estimate the time to reach radioprotection dose limitation and provide safety instruction for patient habits and working environment. The AAPM and Groupe Européen de Curiethérapie/European Society for Radiotherapy & Oncology (GEC/ESTRO) reported the detailed radiation protection measures in BT (53).

Generally, the dose calculation for BT has been based on the AAPM Task Group No. 43 (TG-43). However, there might be some limitations in TG-43. A new algorithm, model-based dose calculation algorithms (MBDCAs), was proposed to compensate for the limitation of TG-43. Enger et al. (54) compared the dose calculation based on the TG-43 and MBDCAs on BT for various tumor types to investigate the influence of the tissue and seed/applicator heterogeneities on BT dose distributions. For prostate or gynecological cancers, the dosimetry influence of MBDCAs was comparable to TG-43, while the impact of MBDCAs might be more pronounced on other sites. Although MBDCAs have the potential to offer dosimetry benefits than TG-43, there is no agreement on how to integrate these advanced BT dose calculation techniques in clinical practice. Sufficient and independent data documenting the dosimetry influence of MBDCAs are required to support the transition from the TG-43 dose calculation formalism to MBDCAs (25, 54).

## Clinical Performance of I-125 BT

**Figure 3:**
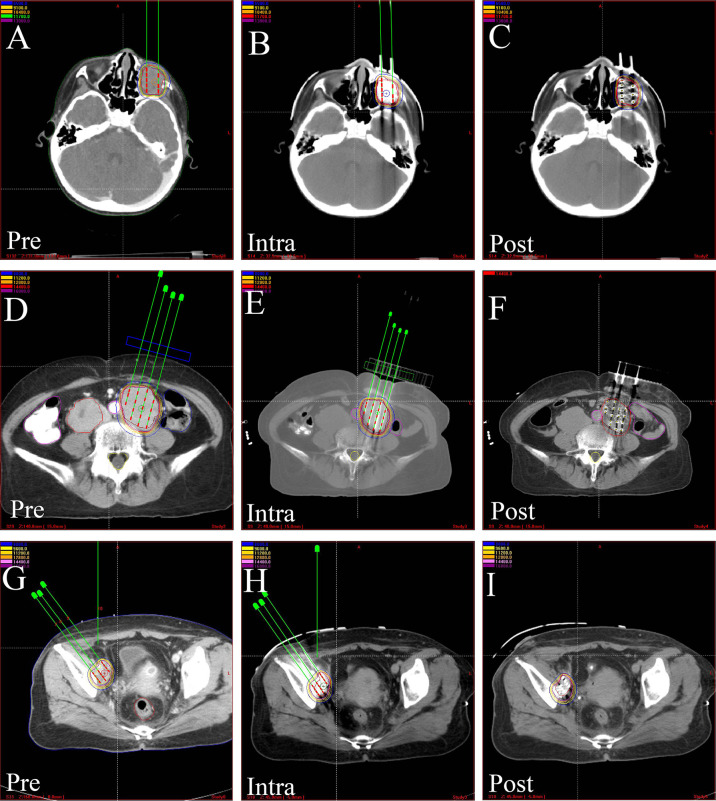
The CT-scan images of treatment plan assisted by 3D-PCT. Preoperative treatment plan, intraoperative verification, and postoperative dosimetric evaluation in, **(A–C)** orbital rhabdomyosarcoma, **(D–F)** kidney cancer, **(G–I)** pelvic recurrence of cervical cancer. 3D-PCT, three-dimensional printing coplanar template.

**Figure 4:**
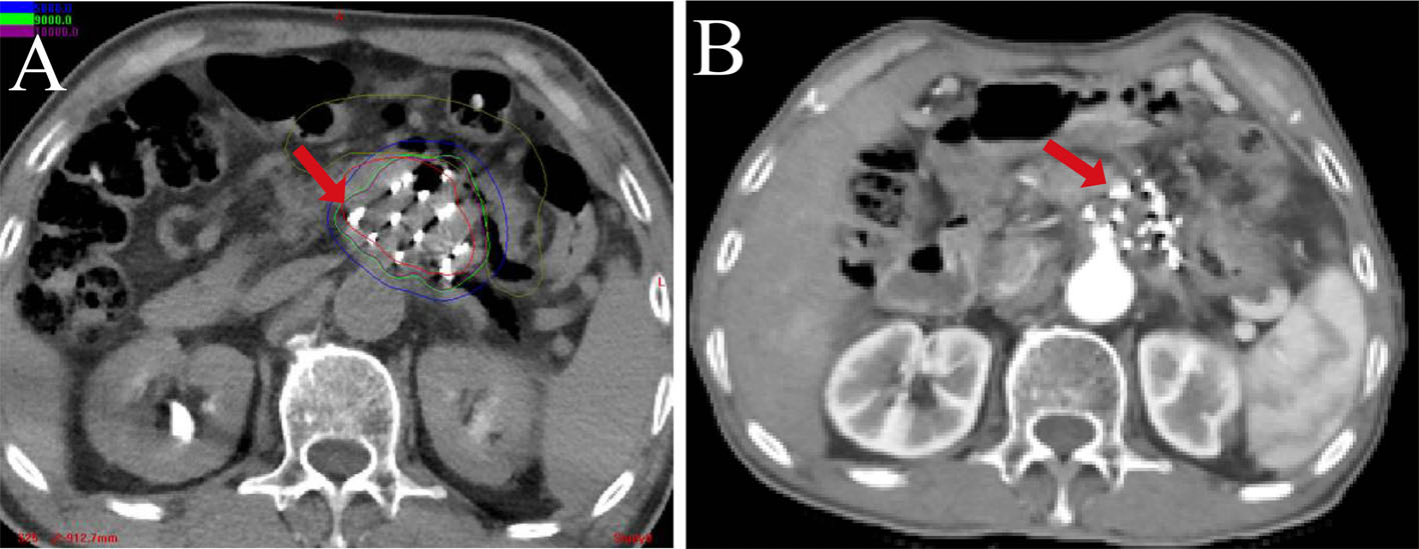
The CT-scan image of 125I-RSI BT in pancreatic head cancer. **(A)** The postoperative actual dose distribution. **(B)** The postoperative follow-up at 6 months.

More recently, I-125 BT has been widely used in the treatment of various tumors (**Table 2**) (55–74) due to its compelling efficacy and potential for more general applicability (**Figures 3**, **4**). The clinical application of I-125 BT is mainly divided into the permanent interstitial implantation of I-125, known as I-125 seed implantation, and the placement of a SEMS loaded with I-125, called an I-125 seed stent. The following showed the clinical efficacy of these two patterns (**Table 3**).

**Table 2:** Patients selection for I-125 LDR-BT and HDR-BT in different anatomical sites (based on ASCO/CCO, ABS, GEC/ESTRO and NCCN guideline).

	LDR-BT	HDR-BT
Breast cancer	Less commonly used than HDR-BT. The patient selection includes tumor size≤3cm, negative surgical margins width≥2mm, distance to skin surface>5mm and fluid cavity≤2.5cm Localization for image-guided surgical excision of non-palpable breast lesions.	Adjuvant APBI or boost after BCS Low-risk group of patients is good candidate, including patients age>50 y with IDC measuring≤2cm, no ILC, no DCIS, tumor size≤30mm(pT1-2), negative margins width≥2mm, unicentric, unifocal, no EIC, no LVI, pN0, no neoadjuvant chemotherapy and any hormone receptor status. Intermediate-risk group of patients is selective candidates, including patients age>40-50 y, tumor size≤30mm(pT1-2), close surgical margins(<2mm), multifocal(limited within 2cm), no EIC, no LVI, pN1mi/pN1a, no neoadjuvant chemotherapy and any hormone receptor status.
Bone metastasis	Unifocal lesion: unresectable or refusing surgery and EBRT; Palliative treatment for multifocal lesions in vital sites; Intolerable to EBRT or recurrence after EBRT.	Few reported.
Lung cancer	NSCLC: unresectable tumor; intolerable to or refusing surgery or chemoradiotherapy; postoperative recurrence and unable to re-surgical excision; incomplete resection through preoperative evaluation; resection margins are macroscopically or microscopically involved; no multiple distant metastasis or controllable metastasis after systematic therapy; KPS>60; life expectancy>6months; diameter≤7cm. SCLC: resistant to chemotherapy and conventional radiotherapy or recurrence after chemoradiotherapy. Lung metastasis/mediastinal lymph node metastasis: unilateral pulmonary lesion≤3, maximum diameter≤5cm; for bilateral lung lesions, each pulmonary lesion≤3, maximum diameter≤5cm. Self-expandable stent loaded I-125 is used for malignant airway obstruction.	Early-stage lung cancer confined to the endobronchial lumen Palliative treatment for locally advanced tumor with central obstructing lesions, particularly in patients who have previously received EBRT or who are not candidates for EBRT or surgical resection. Collapsed lung at the first presentation; Boost to radical EBRT for patients with central tumors.
Pancreatic cancer	Unresectable lesion with life expectancy>3months; Pancreatic metastasis or local lymph node metastasis; Residual lesion after surgery or positive surgical margins. Selective option to relieve epigastric pain for patients with life expectancy < 3 months and tumor diameter>6cm. Self-expandable stent loaded I-125 is used for malignant biliary obstruction.	Few reported.
Gynecological cancer Cervical cancer Endometrial cancer Vaginal cancer	Postoperative recurrence without surgical indication, diameter≤5cm; Recurrent lesion after surgical excision, EBRT or HDR. Pelvic lymph node metastasis.	For cervical cancer: Intact uterus: as a boost modality for locally advanced cancer; Boost to EBRT for early stage, inoperable cancer. Combined with EBRT after surgical excision, in case of positive margins. For endometrial cancer: Boost to EBRT for patients not amenable to surgery; Postoperative adjuvant treatment: FIGO stage IA, grade 1-2 in case of risk factors (age ≥ 60 years and/or LVI); stage IA grade 3 and stage IB grade 1-2; stage II grade 1-2 (± EBRT); combined with EBRT for boosting vaginal vault in HR patients (FIGO stage IB, grade 3) or in advanced disease. For vaginal cancer: Patients with primary stage I-IV vaginal cancers or recurrent cervical, endometrial, or vulvar carcinoma in the vagina with residual vaginal lesions>0.5cm thick.
Hepatocellular carcinoma	For primary lesion: Locally advanced and unresectable lesion; Tumor diameter ≤7cm; No invasion to big vessel; Intraoperative residual lesion or positive tumor margin; Unfavorable efficacy after TACE; combined with TACE; Recurrence after surgical excision and unable or refusing to re-surgery. For metastasis: unifocal lesion diameter <7cm; multifocal lesion<5, maximum diameter≤3cm; unresectable lesion Residual, relapse, or newly diagnosed lesion after surgery or TACE. Child-Pugh A or B Boost after EBRT.	Large(>5cm) or centrally located lesions. Lesions in the liver dome or near the liver capsule Lesions located near vascular or biliary structures.
Prostate cancer	Monotherapy: GS≤6, PSA<10 ng/ml; GS=7, PSA<10 ng/mL or GS=6, PSA=10-20 ng/ml. Boost after EBRT: GS=7, PSA=10-20ng/ml choosing EBRT±ADT. GS≥8, PSA>20ng/ml receiving EBRT and ADT	Monotherapy: GS≤6, and PSA<10ng/ml and T1-T2a tumor. Boost or monotherapy: GS=7, or PSA=10-20ng/ml, or T2b-T2c tumor. Boost: GS=8-10, or PSA>20ng/ml, or T3a tumor.
Esophageal cancer	Self-expandable stent loaded I-125 is used for malignant esophageal obstruction.	Thoracic esophageal lesions ≤10 cm in length; Confined to the esophageal wall; No invasion to adjacent organ or vessel, no regional lymph node metastasis.
Ophthalmic tumor	For melanoma ABS-OOTF recommended that most melanomas of the iris, ciliary body, and choroid could be treated with plaque brachytherapy. Patients with AJCC T1-3, and T4a-d uveal melanoma can be candidates without histological verification, after counseling about likely vision, eye retention, and local control outcomes. Patients with peripapillary and subfoveal and those with exudative retinal detachments should be informed the potential outcomes. Patients with T4e tumors, extraocular extension, a basal diameter exceeding the limits of brachytherapy, blind painful eyes, and no light, are not suitable for plaque therapy. For retinoblastoma Primary brachytherapy for tumor locating at anterior to the equator and in unilaterally affected children. Secondary therapy for residual or recurrent tumor irrespective of location, except anterior segment involvement and juxta papillary location.	Few reported.
Rectal cancer	Locally recurrent and unresectable lesion; EBRT is impracticable, including refusing EBRT or having primary pelvic RT history; Focal boost to EBRT or salvage after chemotherapy; Palliative treatment for hepatic and pulmonary oligometastasis with local recurrence. Appropriate percutaneous puncture pathway for implant	HDR alone Histologically confirmed rectal cancer, staged cT1/cN0, less than 3 cm in greatest diameter. Well to moderately differentiated adenocarcinoma. Tumor configuration of non-ulcerative, polypoid mobile tumor. Lesions above the anal verge≤15cm, with lesion thickness <1 cm Postoperative HDR Uncertain resection margin Rx or involved resection margin (R1) Boosting to EBRT for poorly differentiated adenocarcinoma and lympho-vascular invasion. Palliative treatment for local recurrence
Retroperitoneal lymph node metastasis	Symptomatic or asymptomatic metastasis, diameter≤7cm; Symptomatic or asymptomatic multi-metastasis; Relapse lesion after surgery, EBRT, chemotherapy or molecular targeting therapy; Life expectancy>3 months.	Few reported.
Nonmelanoma skin cancer	Few reported	Primary T1-T2 tumor or T3-T4 after EBRT. Unresectable tumor or recurrent tumor after surgery. Unable to meet cosmetic need by plastic surgery. Superficial mould BT is suitable for tumor≤5mm. Interstitial BT is suitable for tumor thickness>5mm with irregular shape or locating at curved surface.
Central nervous system	1.Primary tumor: unresectable and untreated tumor in any location, with diameter≤5cm; residual tumor after surgical resection; recurrent tumor after surgery or chemoradiotherapy. Brain metastasis: unifocal or multifocal<3.	Few reported.
Soft tissue sarcoma	Locally advanced and unresectable tumor, with diameter≤7cm; Macroscopically or microscopically residual lesions during surgery; Recurrence after surgical excision or EBRT; Metastatic tumor without surgical indication; Boost to surgery+ EBRT regimen for locally advanced tumor; Palliative treatment for locally advanced tumor that is beyond control or distant metastasis along with severe local symptoms.	Monotherapy: Tumor diameter within 5-10cm; negative surgical margins; Low-risk cases CTV adequately cover the target volume Within the dose constrictions of OARs Re-irradiation Pediatrics Combined with EBRT High risk of recurrence (>10 cm, recurrent disease without previous radiation, or positive surgical margins); BT alone beyond the dose limitation of OARs; Petroperitoneum, head and neck; Skin and lymph node are involved.
Primary spine tumor	Tumor involving adjacent vital organs or incompletely surgical excision; Recurrence or residual lesion after surgery; Local residence after EBRT or EBRT failure; Solitary or oligo lesions without surgical indication.	Few reported.
Head and neck squamous cell carcinomas	Alone T1-2N0; 2. Patient decision Tumor location in areas of functional importance Tumor location in areas of cosmetic relevance such as the periorificial zone. Residual lesion or adjuvant therapy after surgery. Unresectable and locally advanced tumor. Salvage for local recurrence. Combined with EBRT Intact T1-2 tumors in patients ineligible for surgery with a substantial risk of lymph node involvement, Advanced T3-4 and/or N + tumors that would require surgical resections with functional or cosmetic impact (such as cheek, base of tongue); Tumors of different locations eligible for primary radiotherapy in whom a brachytherapy boost outweighs the discomfort of an interventional procedure (i.e., soft palate, tonsil, etc.).	Similar to LDR
Penile tumor	Few reported.	Patients with T1b or T2 disease <4 cm in maximum dimension and confined to the glans penis Tumor with minor extension across the coronal sulcus are also suitable, when the extension can be covered with no more than one additional plane of needles.
Bladder cancer	Few reported.	Muscle-invasive bladder carcinoma Solitary tumor of a maximum diameter <5 cm. No concurrent carcinoma in situ elsewhere in the bladder. UICC TNM classification cT2-T3. Tumor not located in the bladder neck or the prostatic urethra (male)5. No distant metastasis.

±, with or without; I-125, iodine 125; LDR-BT, low-dose-rate brachytherapy; HDR-BT, high-dose-rate brachytherapy; ASCOCCO, American Society of Clinical Oncology/Cancer Care Ontario; ABS, American Brachytherapy Society; GEC/ESTRO, Groupe Européen de Curiethérapie / European Society for Radiotherapy & Oncology; NCCN, National Comprehensive Cancer Network; APBI, accelerated partial breast irradiation; BCS, breast-conserving surgery; ADT, androgen deprivation therapy; EBRT, external-beam radiotherapy; FIGO, International Federation of Gynecology and Obstetrics (version 2009); NSCLC, non-small cell lung carcinoma; SCLC, small cell lung carcinoma; IDC intraductal carcinoma; ILC, invasive lobular carcinoma; DCIS, ductal carcinoma in situ; EIC, extensive intraductal component; LVI, lympho-vascular invasion; GS, Gleason score; PSA, prostate-specific antigen; LR, low risk; HR, high risk; TACE, transhepatic arterial chemotherapy and embolization; UICC, Union for International Cancer Control; TNM stage, tumor, node and metastasis stage; OAR, organ at risk; CTV, clinical target volume; ABS-OOTF, American Brachytherapy Society-Ophthalmic Oncology Task Force; AJCC, American Joint Committee on Cancer.

**Table 3:** The key controlled clinical trials of I-125 seeds therapy in the past decade.

Methods	Study	Year	N	Tumor Type	Interventions	Outcomes	Ref
I-125 seeds implantation	Liu	2020	83	NSCLC recurrence after first-line CT failure	MWA *vs.* I-125-BT	Median OS time: 30.57 *vs.* 31.63 months; P=NR. DFS rate: p=0.153;1-year 92.16% *vs.* 93.75%;2-year 76.47% *vs.* 78.13%. Pleural effusion: 3.92% *vs.* 3.13%, P < 0.05.	(75)
	Xiang	2019	95	Bilateral lung recurrences from HCC	I-125-BT *vs.* supportive treatments	Median PFST time: 20.4 vs 14.3 months, P= 0.026; Median OS time: 23.0 vs 15.6 months, p<0.01.	(76)
	Pang	2019	184	Advanced cholangiocarcinoma	PTBS+ I-125-BT *vs.* PTBS	Median OS time: 13 *vs.* 8 months, P<0.001. Biliary re-obstruction: 35.2% *vs.*19.5%, P = 0.017	(56)
	Tsubokura	2018	757	Prostate cancer	IG-IMRT *vs.* I-125 BT	5-y BFFS rate: 88.7% *vs.* 96.7%, P=0.0003 5-y OS rate: 98.5% *vs.* 98.4%, P=0.0139.	(77)
	Lee	2018	687	Prostate cancer	EBRT+I-125 BT *vs.* EBRT	3-y OS rate: 97% *vs.* 95.3%, P=0.26 3-y CSS rate:100% *vs.* 99.5%, P=0.56	(78)
	Luo	2018	320	Prostate cancer	MAB + EBRT + I-125 BT *vs.* MAB + EBRT	OS; 12.3 vs 9.1 years, P < 0.001; BRFS; 9.8 vs 6.5 years, P < 0.001; SRE: 10.4 vs 8.2 years, P < 0.001; CCT; 11.6 vs 8.8 years, P = 0.007.	(79)
	Kishan	2018	1809	Prostate cancer	RP *vs.* EBRT *vs.* EBRT+BT	5-y prostate CSM: 12% *vs.* 13% *vs.* 3% 5-y incidence of DM: 24% *vs.* 24% *vs.* 8% 7.5-year all-CM: 17% *vs.* 18% *vs.* 10%	(80)
	Wu	2018	50	Stage III/IV NSCLC	I-125-BT + TP *vs.* TP	Median OS time: 20 *vs.* 15 months, P<0.05; Median PFS time: 13 *vs.* 8 months, P<0.05.	(57)
	Sun	2018	134	HCC	I-125-BT +TACE *vs.* TACE	Median OS time: 11 *vs.* 5.8 months; p=0.006;	(69)
	Li	2018	54	HCC	I-125-BT +TACE *vs.* TACE	Median OS time: 11 *vs.* 9 months, P=0.022. Median biliary patency time: 6 *vs.* 4 months, P=0.001.	(59)
	Mo	2018	93	Metastatic soft tissue sarcoma after first-line CT failure	I-125-BT +gemcitabine *vs.* gemcitabine	Mean PFS time: 7.1 *vs.* 3.6 months; P<0.001; Mean OS time: 16.9 VS. 12.1; P=0.107.	(81)
	Johnson	2017	25038	Prostate cancer	I-125-BT-Boost *vs.* EBRT	7-yr OS: 82% vs 73%; p < 0.001	(82)
	Tu	2017	116	Locoregional HCC Recurrence	I-125-BT +RFA *vs.* RFA	Mean TTR time: 21.7 *vs.* 15.9 months, P=0.733; Mean OS time: 41.7 *vs.* 40.9 months, P= 0.316.	(83)
	Zheng	2017	66	Pancreatic head cancer	Surgery+ I-125-BT *vs.* surgery	Mean PFS time: 8 *vs.* 5 months, P<0.001; Median OS time: 11 *vs.* 7 months, P<0.001.	(84)
	Yan	2017	81	Locally recurrent NPC after EBRT with or without CT	I-125-BT *vs.* Re-IMRT	Median LTPFS time: 21 *vs.* 17 months; P=0.015; Median OS time: 25 *vs.* 24 months; P=0.346.	(85)
	Huang	2016	210	HCC with PVTT	I-125-BT +TACE *vs.* TACE	Median OS times: 11.0 and 7.5 months, P<0.001; Survival rate: p <0.001; 12-month 50% *vs.* 25 %; 24-month 14.5% *vs.* 9 %; 36-month14.5 % *vs.* 5 %.	(62)
	Li	2016	144	HCC of 3 to 5cm	I-125-BT +TACE *vs.* TACE	Median OS time: 30 *vs.* 18 months, P<0.001; Survival rate: P<0.001; 1-year 89.1% *vs.* 65.5%;3-year 51.0% *vs.* 7.4%;	(63)
	Yan	2016	65	Distant metastases in the oral cavity and maxillofacial region	I-125-BT *vs.* Re-IMRT	Local control rates: P<0.05; 3-month 83.9% *vs.*76.5%; 6-month 75% *vs.*62.5%; 12-month 66.7% *vs.*43.8%; 18-month 38.4% *vs.* 25.0%; 24-month 25.0% *vs.*0.0% Median LTPFS time: 14 *vs.* 9 months, P<0.001.	(86)
	Luo	2016	276	HCC with main portal vein tumor thrombus	I-125-BT +TACE *vs.* TACE	Median OS time: 9.3 *vs.* 4.9 months, P<0.001; Median PFS time: 1.8 *vs.* 1.5 months, P<0.001. Median stent Patency Period: 9.2 *vs.* 4.8 months, P<0.001	(64)
	Wang	2016	72	Bilateral lung recurrences from colorectal carcinoma	I-125-BT *vs.* supportive treatments	Mean OS time: 18.8 *vs.* 8.6 months, P=0.008	(87)
	Morris	2015	398	Prostate cancer	I-125-BT *vs.* EBRT	bDFS: P<0.001 3-year 94% *vs.* 94%;5-year 89%*vs.* 84%;7-year 86% *vs.* 75%;9-year 83% *vs.* 62% Grade 3 GU Toxicity: 19% *vs.* 5%, P<0.001.	(70)
	Yu	2015	52	Stage III NSCLC after con-CRT	I-125-BT+DP *vs.* DP	PFS time: 8 *vs.* 5.5 months; P < 0.05; LCR time: 10 *vs.* 6.2 months; P< 0.05.	(65)
	Li	2015	71	Unresectable stage III/IV NSCLC	I-125-BT *vs.* EBRT	Median OS time: 16 *vs.* 10 months, P<0.01	(66)
	Chen	2014	136	HCC	I-125-BT +RFA *vs.* RFA	Mean OS time: 95.8 *vs.* 70.8 months P=0.003. Survival rate: P=0.003; 1-year 100% *vs.* 95.6%; 2-year 95.6% *vs.* 85.2%; 3-year 86.7% *vs.* 75.0%; 4-year 73.5% *vs.* 58.8%; 5-year 66.1% *vs.* 47.0% Recurrence rate: (P=0.004): 1-year 4.5% *vs.*14.8%; 2-year 11.8% *vs.* 25.0%; 3-year 22.1% *vs.* 35.3%; 4-year 32.4% *vs.* 47.1%; 5-year 39.8% *vs.* 57.4% Local recurrence rate: 7.3% *vs.* 22.0%, P=0.012; Intrahepatic recurrence rate: 17.6% *vs.* 32.3%, P=0.041.	(88)
	Yang	2014	85	HCC with PVTT	I-125-BT+TACE *vs.* TACE	Median OS time: 210 *vs.* 154.0 days, P=0.00.	(67)
	Hu	2012	73	Recurrent Glioma	I-125-BT*vs.* CRT	Median OS time: 29.3 *vs.* 19.7 months, P<0.05.	(89)
	Zhang	2011	53	Advanced NSCLC	I-125-BT+GP *vs.* GP	Median OS time: 13.5 *vs.* 9.0months, P=0.260; PFS time: 8.0 *vs.* 5.0 months, P=0.048.	(71)
I-125 seeds loaded stent	Wang	2019	32	Pancreatic cancer	I-125 stent *vs.* conventional stent	Median OS time: 10.4 *vs.* 9.7 months, P=0.027. Median stent patency time: 9.8 vs 8.8 months; P=0.019.	(55)
	Wu	2018	111	HCC+MPVTT	I-125-BT +TACE+stent *vs.* TACE +stent	Survival rate: P<0.05: 6 month 85.2 *vs.* 50.9; 12 month 42.6 *vs.* 10.5; 24 month 22.2 *vs.* 0. Restenosis rate (P<0.05): 6 month 18.5% *vs.* 43.9%; 12 month 55.6%*vs.* 82.6%; 24 month 83.3% *vs.* 96.5%	(58)
	Wang	2018	66	Malignant Airway Obstruction	I-125 stent *vs.* conventional stent	Median OS time: 170 *vs.* 123 days, P<0.05. Restenosis rate (P=0.037): 21.2% *vs.* 45.45%	(14)
	Zhu	2018	328	Malignant biliary obstruction	I-125 stent *vs.* conventional stent	Median OS time: 202 days *vs.* 140 days, P = 0.020; Relief of jaundice: 85% *vs.* 80%; P = 0.308. Restenosis rate (P=0.010): 90 days 9% *vs.* 15%; 180 days 16% *vs.* 27%; 360 days 21% *vs.* 33% Grade 3/4 complications rate: 8.5% *vs.* 7.9%; P =0.841	(12)
	Yang	2016	61	HCC with IVCTT	TACE+I-125-stent *vs.* TACE+ bare stent	Median OS time: 203.0 *vs.* 93.0, P=0.006; Propensity score matching OS: 200 *vs.* 66 days, P=0.019.	(61)
	Zhu	2014	148	Esophageal cancer	I-125 stent *vs.* conventional stent	Median OS time: 177 *vs.* 147 days, P=0.0046.	(11)
	Zhu	2012	23	Malignant biliary obstruction	I-125 stent *vs.* conventional stent	Median OS time: 7.40 *vs.* 2.50 months, P=0.006.	(15)
	Guo	2008	53	Esophageal cancer	I-125 stent *vs.* conventional stent	Median OS time: 7 *vs.* 4 months, P<0.001; Median onset of restenosis: 4.5 *vs.* 2.0 months, P<0.044.	(13)

NR, no report; NSCLC, non-small cell lung cancer; CT, chemotherapy; MWA, microwave ablation; RFA, radiofrequency ablation; OS, overall survival; DFS, disease-free survival; HCC, hepatocellular carcinoma; PFST, progression-free survival time; PTBS, percutaneous transhepatic biliary stenting; TP regimen, paclitaxel/cisplatin; MPVTT, main portal vein tumor thrombus; TACE, transcatheter arterial chemoembolization; TTR, time to recurrence; NPC, nasopharyngeal carcinoma; EBRT, external beam radiotherapy; IMRT, intensity-modulated radiotherapy; LTPFS, local tumor progression-free survival; IVCTT, inferior vena cava tumor thrombosis; LC, local control; CRT, chemoradiotherapy; DP regimen, docetaxel/cisplatin; GP regimen, gemcitabine/cisplatin.

## I-125 Brachytherapy in Solid Tumors

### Brain Tumor

On account of lesions’ deep localization, high eloquent areas, and lesion size, additional strategies are required to complement surgery in the case of incomplete resection of a brain tumor (90). Moreover, the limited efficacy on large lesions and the increased risk of radiation necrosis also hinder the application of radiosurgery on brain metastasis (91). Several studies have confirmed the efficacy of I-125 BT in treating cerebral lesions (92). For recurrent gliomas, a controlled trial (n = 73) demonstrated that the use of I-125 BT significantly improved the median OS (29.3 *vs.* 19.5, p < 0.05) and the 2-year OS rate (77.1 *vs.* 42.5, p < 0.05) when compared to traditional chemoradiotherapy (89). In addition, a study enrolling 147 patients with inoperable or residual low-grade gliomas after incomplete resection that received stereotactic I-125 seed implantation reported that 5-year and 10-year survival rates were 93% and 82%, respectively, with only a tumor volume of >15 ml as a risk factor for recurrence (90). With respect to WHO grade II and III gliomas, stereotactic I-125 BT is likely a safer and more feasible treatment compared to surgical resection due to its minimally invasive nature and low risk for procedural complications. A single-arm study assessed the efficacy of stereotactic I-125 BT in 172 patients with primary and recurrent grade III anaplastic gliomas, demonstrating the OS and progression-free survival (PFS) in the *de novo* cohort (median OS of 28.9 and median PFS of 21.4 months) and recurrence cohort (median OS 49.4 and median PFS of 32.6 months) (93). A retrospective study provided class IV evidence on the efficacy of resection in conjunction with I-125 BT on brain metastasis (91). Therefore, I-125 BT has freshened the possibility of increased quality of life for patients and longer survival for multiple WHO grade brain tumors, both primary and recurrent, as well as for intracranial metastasis, however, in the presence of highly selective indications. To integrate I-125 BT into the guidelines for brain tumor treatment, RCTs are needed.

### Eye Tumor

For uveal melanoma (UM), the Collaborative Ocular Melanoma Study (COMS) reported no significant difference in survival rates between the patients who were treated by enucleation and plaque BT (94). Moreover, several studies demonstrated that plaque BT has a comparable mortality rate for enucleation over 20 years of follow-up (95). Additionally, plaque BT showed a comparable outcome with proton beam radiotherapy regarding 5-year mortality and LC rates (96). One single-arm study (n = 677) published last year reported the long-term patient survival rate after I-125 BT of UM with regard to relative survival rates of 74% at 5 years, 64% at 10 years, 62% at 20 years, 83% at 30 years, and ≥100% at 32 to 40 years (97). Thus, I-125 plaque BT has become the commonly used modality for UM because of its favorable clinical outcomes and offering an opportunity for globe-sparing. A multicenter retrospective cohort study evaluated the impact of gene expression profile class designation on the UM height response to I-125 plaque BT, indicating that Class 1 UM regress more rapidly than Class 2 UM in the first 6 months after I-125 plaque BT (p = 0.007) (98). A novel nomogram was developed to predict the visual acuity outcome for UM after I-125 plaque BT (99). However, due to the risk of radiation retinopathy, plaque BT always acts as rescue or salvage treatment before enucleation (100). I-125 is generally used in the USA, while Ru-106 and Pd-103 are more frequent in Europe and others (101). Moreover, it was shown that compared with Ru-106, I-125 had significantly less rates of repeated BT among patients with bulky choroidal melanomas, without significant difference in enucleation rate or patient survival (102). A recent study showed that US-guided I-125 plaque BT has reduced the failure rate from 9.3% to 1.5% (103). Furthermore, I-125 plaque BT also has excellent outcomes in residual or recurrent iris melanoma after surgical resection (tumor control at 6 years of 87%) (104), juxtapapillary choroidal melanoma (80% of 10-year LC) (105), scleral-invasive conjunctival squamous cell carcinoma (LC rate of 100%) (106), and selected retinoblastomas that fail chemoreduction (95% tumor control) (107). However, radiation-induced adverse reaction associated with the brain and vision is also of concern. The efficacy of I-125 BT in eye disease requires more investigation (108).

### Head and Neck Tumor

The complicated anatomical structure of the head and neck renders certain cases inoperable. The typical example is nasopharyngeal cancer (NPC), for which chemoradiation is the treatment of choice. According to the latest studies, a regimen of gemcitabine plus cisplatin in induction chemotherapy for locoregionally advanced NPC has significantly increased the OS and PFS time (109). However, this regimen is not suitable for all head and neck cancers due to diverse lesion sites and tissue types. In the consideration of cosmetic and functional outcomes, I-125 BT is an appropriate alternative (110). A clinical trial (n = 81) evaluating the efficacy of I-125 BT in locally recurrent NPC when compared with external beam re-radiotherapy reported a higher median local tumor PFS (LTPFS; 21 *vs.* 17 months, p = 0.015), without meaningfully impacting OS (85). Another study reported that the median LTPFS time (14 *vs.* 9 months, p < 0.001) of I-125 BT in treating distant metastases in the oral and maxillofacial region compared to EBRT was also higher (86). Moreover, several single-arm clinical studies suggested that I-125 BT performed exceptionally well in various head and neck cancers, including primary mucoepidermoid carcinoma of the parotid gland (10-year OS rate of 95.8%), adenoid cystic carcinoma (ACC) involving the skull base (3-year OS rate of 62.6% and 3-year PFS rates of 46.4%), and salivary gland carcinomas of the lip and buccal mucosa (10-year LC rate of 82.9% and 10-year OS rate of 77.8%) (111–113). One study reported that the use of I-125 BT in non-squamous carcinoma results in a better outcome compared to squamous cell carcinoma (2). Since preliminary results indicate the efficacy of I-125 BT for head and neck cancer, a trial with a larger sample size and a longer follow-up is recommended.

### Lung Cancer

The majority of lung cancer patients present with an advanced stage at initial diagnosis. Consequently, multimodality therapy is considered the principal strategy for the treatment of lung cancer. As radiology and technology are still progressing, CT-guided I-125 BT has now become a choice for inoperable lung cancer (114, 115). A single-center RCT conducted in China (72 patients with bilateral recurrence from colorectal cancer) showed that the CT-guided I-125 BT was equally well tolerated but had a significantly prolonged survival (mean 18.8 vs. 8.6 months, p = 0.008) when compared with standard palliative care, without an increase in complications (87). Similarly, a trial of 95 patients with bilateral lung recurrences from hepatocellular carcinoma (HCC) after resection or ablation demonstrated a greater PFS (median 20.4 *vs.* 14.3 months, p = 0.026) and OS (median 23.0 *vs.* 15.6 months, p < 0.01) when comparing I-125 BT with chemotherapy (76). A meta-analysis (updated in 2018) included 15 clinical trials and confirmed that the combination of I-125 BT with chemotherapy or even I-125 BT alone improves OS of non-small cell lung cancer (NSCLC) patients when compared with chemotherapy alone [pooled hazard ratio (HR): 0.66, 95% CI: 0.50–0.86, p<0.001], albeit at the expense of additional, yet manageable, myelosuppression (116). However, even though I-125 BT is more effective compared to standard palliative care, there are still limitations to I-125 BT. First, a large multicenter RCT is needed to distinguish differences in the clinical outcome of I-125 BT, chemotherapy alone, chemotherapy with EBRT, and EBRT alone. Second, a combination of I-125 BT with molecular targeted therapy should be considered for study to improve long-term efficacy. Finally, as an alternative for the treatment of NSCLC recurrence, I-125 BT is equally effective and safe as microwave ablation (MWA) (75).

### Hepatocellular Carcinoma

Hepatic resection is appropriate for only a few patients with hepatocellular carcinoma (HCC) because preexisting liver dysfunction and the presence of HCC in the major vessels of the liver may exclude the possibility of liver resection. Because donor shortages restrain liver transplantation and because of the suboptimal long-term efficacy of radiofrequency ablation (RFA) with or without transhepatic arterial chemotherapy and embolization (TACE), clinicians have explored better strategies (117). A prospective RCT that included 136 patients with HCC [all had hepatitis B virus (HBV) infections] compared RFA plus I-125 BT with RFA alone. RFA combined with I-125 BT when compared with RFA alone resulted in lower recurrence rates at 1, 2, 3, 4, and 5 years (4.5% *vs.* 14.8%, 11.8% *vs.* 25.0%, 22.1% *vs.* 35.3%, 32.4% *vs.* 47.1%, and 39.8% *vs.* 57.4%; p = 0.004), lower local and intrahepatic recurrence rates (7.3% *vs.* 22.0%, p = 0.012; 17.6% *vs.* 32.3%, p = 0.041), and higher survival rates at 1, 2, 3, 4, and 5 years (100% *vs.* 95.6%, 95.6% *vs.* 85.2%, 86.7% *vs.* 75.0%, 73.5% *vs.* 58.8%, and 66.1% *vs.* 47.0%; p = 0.003) (88). However, a study that evaluated whether I-125 BT as a prophylactic after RFA could benefit patients with locoregional HCC recurrence in terms of time to recurrence (TTR) and OS indicated no difference with and without I-125 BT in TTR (21.7 *vs.* 15.9 months, p = 0.733) and OS (41.7 *vs.* 40.9 months, p = 0.316) (83). Therefore, further study is needed to prove the efficacy of I-125 BT as a prophylactic after RFA. Additionally, two meta-analyses suggested that I-125 BT plus TACE was found to remarkably improve the survival of patients with HCC when combined with portal vein tumor thrombus (PVTT) compared to TACE alone (118, 119).

### Pancreatic Cancer

The implantation of radium in treating pancreatic cancer dates back to the 1930s, almost 40 years after Marie and Pierre Curie discovered radium (120). In 1981, the first reported US-guided I-125 BT was also performed on pancreatic cancer (8). In other words, the use of seed implant BT in pancreatic cancer has occurred since the earliest phases of seed implant BT development. A controlled study (66 patients with pancreatic head cancer) suggested a significantly longer PFS (8 *vs.* 5 months, p < 0.001) and OS (11 *vs.* 7 months, p < 0.001) for surgical bypasses plus I-125 BT compared to biliary and gastric bypass alone (84). Additionally, a recent review summarized the development of I-125 BT on pancreatic carcinoma using several approaches, such as with *in vitro* studies and preclinical studies, evaluating the complications and efficacy of I-125 BT alone or in combination with another strategy (cryoablation, bypass surgery, chemotherapy, and EBRT) (121). It pointed out that a uniform dose and standardized procedure are necessary to achieve homogeneity and provided guidance for clinical practice (121). Given the high mortality rate and poor prognosis of pancreatic cancer with current treatment modalities, the combination of I-125 BT with other therapies is a hot topic for future research.

### Prostate Cancer

LDR-BT is an acceptable option for selected patients with prostate cancer for any risk group (77–79, 82). According to the ABS guidelines, the GEC/ESTRO guideline and American Society of Clinical Oncology/Cancer Care Ontario (ASCO/CCO) Joint guideline, LDR-BT could be utilized as monotherapy for low-risk and low–intermediate-risk prostate cancer; in addition, LDR boost or HDR boost is recommended for patients with selectively intermediate–high-risk and high-risk prostate cancer (7, 26, 122–124). The recommendation was formulated based on several randomized controlled trials comparing EBRT, LDR boost, and HDR boost. One of them, ASCENDE-RT RCT (n = 398, low-intermediate = 2; high-intermediate = 120; high = 276), which was highly anticipated, demonstrated that LDR boost after EBRT significantly improved biochemical control in men with intermediate- and high-risk cancer compared with EBRT alone [biochemical disease-free survival (bDFS); 83% *vs.* 62% of 9-year bDFS, p < 0.001]. However, increased Grade 3 GU toxicity was observed in the I-125 LDR group compared with EBRT alone (19% *vs.* 5%, p < 0.001) (125). In other words, LDR boost improves LC at the cost of increasing GU or GI toxicity. Furthermore, in this guideline, no meaningful difference was shown regarding the dosimetric advantage of 125I seeds compared to other isotopes. Although ABS did not recommend a specific radioactive source for LDR treatment, I-125 is indeed the most commonly used radioactive source in clinical practice due to its appropriate energy and half-life. Furthermore, the OS advantage for I-125 BT compared with EBRT remains to be determined when accounting for the fact that most trials are powered for PFS (7).

Since both LDR and HDR are recommended as monotherapy or boost for selective men with prostate cancer, the question that which is the more favorable BT modality remains controversial, as it needs comprehensive analysis of various oncological indexes including OS rate, LC, biochemical control, GU/GI toxicity, and even quality of life (**Tables 4**, **5**) (129, 131–134). In the past few years, an increasing number of studies compared the clinical efficacy of LDR and HDR from various aspects. From the perspective of radiobiology, the rapid dose delivery seen in HDR is deemed to achieve a more destructive effect on lower α/β ratio cells, such as prostate cancer cells. The real difference in tumor control needs to be further confirmed through clinical observation (135).

**Table 4:** The comparison of oncological results of LDR and HDR.

Study	Year	N	Tumor type	Interventions	Outcome	Toxicity	Ref
Matthew	2020	54642	Prostate cancer	EBRT mono (n=51547) HDR-BB (n=2765) LDR-BB (n=330)	5-y PCSM, p=0.020 3.5% *vs.* 2.7% *vs.* 2.7%	5-y GI, p<0.001, 18.7% *vs.* 16.7% *vs.* 32.2% GU, p<0.001,10.4% *vs.* 16.6% *vs.* 15.8% SKE, p=0.041,2.8% *vs.* 2.4% *vs.* 2.7%	(126)
Levin-Epstein	2020	3502	Prostate cancer	SBRT, n=1716; LDR, n=1274; HDR, n=512.	nPSA <0.2 ng/mL, P<0.001, 48% *vs.* 72% *vs.* 56% nPSA <0.5 ng/mL, P<0.001, 80% *vs.* 86% *vs.* 77%		(127)
King	2019	122896	Prostate cancer	HDR-Boost, n=8526 LDR-Boost, n=9877 DE-EBRT, n=104486	Similar OS for HDR *vs.* LDR, AHR 1.01 [0.93, 1.10]; P=0.77		(128)
Slevin	2019	287	Prostate cancer	LDR–EBRT: 116 HDR–EBRT: 171	5-y bPFS p=0.01, 90.5% *vs.*77.6%	Late GU G≥3, p=0.17 8% *vs.* 4% Late GI G≥3, p=0.13 5% *vs.* 1%	(129)
López	2019	119	Prostate cancer	LDR,n = 44; HDR, n = 75	5-y PSA-RFS, P=0.063,79% *vs.* 65% 5-y CSS: P = 0.44, 97% *vs.* 93%	G3 GU, P=0.756 27% *vs.* 22%	(130)
Yamazaki	2018	838	Prostate cancer	HDR-BT mono n=352 LDR-BT ± EBRT n=486	5-y bNED, P=0.25, 95.6% *vs.* 92.9% 7-y CSS, p = 0.07, 100% *vs.* 99.1% 7-y OS rate, p=0.2873, 97.8% *vs.* 93.7%	Accumulated GU G≥2 p = 0.3289 17.6% *vs.* 15.8% Accumulated GI, p = 0.1511 2.8% *vs.* 1.9% Acute GI: G≥1/G≥2, p<0.0001 69.3%/12.3% *vs.* 92%/43% Late GI G1/G2/G3, p = 0.2526 9%/3%/0.3% *vs.* 7%/2%0% Late GU G1/G2/G3, p = 0.0007 28%/16%/3% *vs.* 40%/15%/0.8%	(131)
Kollmeier	2017	98	Prostate cancer	LDR, n=37; HDR, n=61	3-year PSA RFS: 60.2% p=0.84	Late G3/4 GU: 3% *vs.* 13%	(132)
Tward	2016	118199	Prostate cancer	LDR, n=12801 HDR, n=685 LDR+EBRT, n=8518 HDR+EBRT, n=2392		8-y late GU G3, p=NR 15.7% *vs.* 17.4% *vs.* 22.2% *vs.* 26.6%	(133)
Henríquez	2014	56	Prostate cancer	LDR, n=37; HDR, n=19	5-year FFbF: 7%	GU G3/G4: 24%/0% *vs.* 21%/ GI G3/G4: 0%/2.7% *vs.*2%/	(134)

LDR-BT, low-dose-rate brachytherapy; HDR-BT, high-dose-rate brachytherapy; LDR-BB, low-dose-rate brachytherapy boost; HDR-BB, high-dose-rate brachytherapy boost; PSA, prostate specific antigen; nPSA, nadir PSA; RFS, relapse-free survival; CSS, cause-specific survival; bNED, biochemical failure-free survival rates; PCSM, Prostate cancer-specific mortality; SKE, Skeletal-related event; GI, gastrointestinal; GU, genitourinary; OS, overall survival; DFS, disease free survival; LR, local recurrence; LC, local control; FFbF, freedom from biochemical failure.

**Table 5:** Ongoing studies comparing I-125 BT with HDR-BT.

Study identifier	Cancer type	Phase	Study arms	Outcomes	Study Status
NCT02628041	Prostate cancer	II	LDR-BT with I-125 HDR-BT	QOL, LC, urinary function, GU/GI toxicity and PSA nadir value.	Active, not recruiting
NCT01936883	Prostate cancer	III	LDR-BT with I-125 HDR-BT	QOL, PSA RFS.	Recruiting
NCT02258087	Prostate Cancer	II-III	LDR-BT with I-125 HDR-BT	Acute/chronic side effects, QOL, biochemical RFS, locoregional tumor free survival, disease specific survival.	Recruiting
NCT03426748	Prostate Cancer	NA	LDR-BT with I-125 HDR-BT	QOL, time to return to baseline +/- 3 points for the IPSS, acute/chronic toxicity, biochemical outcome, histologic outcome, cell cycle progression score, tumor oxygenation and cell cycle distribution.	Recruiting
NCT02692105	Prostate Cancer	III	LDR-BT with I-125 HDR-BT	QOL, time to return to baseline +/- 3 points for the IPSS, acute/chronic toxicity, TRUS-MRI fusion, biochemical outcome, histologic outcome, cell cycle progression score.	Recruiting
NCT03322280	Hepatocellular Carcinoma	NA	TACE with I-125 TACE	OS, Time to tumor progression, LC, Duration of portal patency, adverse events.	Recruiting
NCT03964064	Pancreatic Cancer	NA	SBRT LDR-BT with I-125	OS, PFS, LC, pain score, QOL, adverse reactions.	Recruiting
NCT02048254	Salivary gland cancer	III	IMRT LDR-BT with I-125	LC, PFS, OS, QOL, radiation-related adverse reactions.	Unknown or recruiting
NCT00664456	Prostate cancer		9 x LHRH-A+I-125 LDR-BT+ 3 x LHRH-A 3 x LHRH-A+I-125 LDR-BT	OS, Clinical PFS, DSS, Salvage therapy non-adaptive interval, QOL, adverse events.	recruiting
NCT03944408	Malignant Airway Obstruction	NA	Metal bare stent with 125I seeds Metal bare stent	Stenosis grade (1 month and 3 months), OS, technical success, complications and side effects, tumor growth rate, the time of emergency endoscopic treatment.	Not yet recruiting
NCT02960087	Prostate cancer	NA	LDR-BT with I-125 HDR-BT	PSA values by 48months, DFS, Adverse events, QOL, Economic analysis.	Recruiting
NCT04610372	Oligometastatic Prostate Cancer	NA	EBRT HDR-BT LDR-BT with I-125 SBRT	Urinary Symptoms, EPIC urinary domain, EPIC bowel domain, EPIC sexual domain, Biochemical failure, Distant metastatic failure, Nodal progression, OS, CSS, Cost effectiveness.	Recruiting

QOL, quality of life; PSA RFS, prostate-specific-antigen recurrence free survival; NA, not applicable; GI, gastrointestinal; GU, genitourinary; IPSS, International Prostate Symptom Score; EPIC, Expanded Prostate Cancer Index; LDR-BT, low dose rate brachytherapy; HDR-BT, high dose rate brachytherapy; I-125, iodine 125; OS, overall survival; PFS, progression-free survival; DSS, disease specific survival; CSS, cancer specific survival; LC, local control; LHRH, a chemotherapy regimen, goserelin acetate 3.6mg/4 weeks or leuprorelin acetate 3.75mg/4 weeks; TACE, transhepatic arterial chemotherapy and embolization.

First, for low-risk disease, multiple studies reported that LDR and HDR have comparable biochemical control as monotherapy for favorable localized prostate cancer (136, 137). Then, for unfavorable diseases, emerging evidence revealed the clinical outcome of HDR and LDR as a boost after surgery or EBRT. A large cohort study, enrolling 122,896 patients who were diagnosed with NCCN intermediate- or high-risk prostate cancer, suggested that HDR boost yields similar OS benefits with I-125 BT boost (p = 0.38) and significantly better OS than dose-escalated EBRT (DE-EBRT) (p < 0.001) (128). A retrospective cohort study enrolled 1,809 patients with Gleason score 9–10 prostate cancer from 12 centers during 2000–2013, which compared the difference of radical prostatectomy (RP), EBRT, and EBRT+BT (LDR-BT or HDR-BT) regarding 5-year prostate cancer-specific mortality rates, 5-year incidence rates of distant metastasis, and 7.5-year all-cause mortality rates (80). Results of that study suggested that EBRT+BT was associated with significantly lower prostate cancer-specific mortality [cause-specific HRs of 0.38 (95% CI, 0.21–0.68) and 0.41 (95% CI, 0.24–0.71)], distant metastasis [propensity score-adjusted cause-specific HRs of 0.27 (95% CI, 0.17–0.43) for RP and 0.30 (95% CI, 0.19–0.47) for EBRT], and all-cause mortality rates [cause-specific HRs of 0.66 (95% CI, 0.46–0.96) for RP and 0.61 (95% CI, 0.45–0.84) for EBRT] than either RP or EBRT alone. However, no significant differences in prostate cancer-specific mortality, distant metastasis, or all-cause mortality were found between the two BT techniques. For Gleason score 10 prostate cancer, a multi-institutional consortium study reported a similar clinical outcome that EBRT+BT yielded superior distant metastasis-free survival (DMFS) compared with RP (p = 0.06) and EBRT alone (p = 0.048) (138). No further comparison between I-125 BT and HDR-BT in this study. However, with regard to prostate-specific antigen (PSA) kinetics, which is an indicator of biochemical control following various modalities for prostate cancer, Levin-Epstein et al. (127) demonstrated that LDR-BT gives rise to lower nadir PSAs (nPSAs) and longer continued decay compared to SBRT and HDR-BT, without significant differences in biochemical recurrence-free survival (RFS). They also noted that whether the difference in prostate-specific antigen (PSA) kinetic implicates the efficacy when LDR and HDR were used as a boost to EBRT requires further investigation (127). A national population-based study compared the GI, GU, skeletal-related events (SREs), and prostate cancer-specific mortality (PCSM) at 5 years in 54,642 patients with intermediate-risk, high-risk, and locally advanced prostate cancer. According to the study, 5-year GI toxicity was significantly lower in the LDR-BB group (32.2%) compared to EBRT (18.7%) and HDR-BB (16.7%) group (p < 0.001). Comparable GU toxicity was seen in both LDR-BB (15.8%) and HDR-BB (16.6%) groups but significantly higher than EBRT (10.4%, p < 0.01). Similarly, comparable prostate cancer-specific mortality was seen in both LDR-BB (2.7%) and HDR-BB (2.7%) groups but significantly lower than EBRT (3.5%, p = 0.02). No significant difference in the SRE was found among these three techniques (p = 0.041) (126). However, for locally recurrent prostate cancer, Henríquez López et al. (130) reveal the comparable efficacy and toxicity between LDR-BT and HDR-BT in terms of 5-year PSA RFS rate (79% vs. 65%, p = 0.063), cause-specific survival (97% vs. 93%, p = 0.44), and grade 3 GU toxicity (27% vs. 22%, p = 0.756). Thus, LDR-BT and HDR-BT yield a similar OS rate and PFS rate among low-, intermediate-, high-risk prostate cancer and locally recurrent prostate cancer, while LDR-BT has lower nPSA and higher GU toxicity. This discrepancy might be due to the longer treatment period of LDR-BT.

Focal therapies for prostate cancer, including RP, cryotherapy, high-intensity focused ultrasound (HIFU), laser ablation, photodynamic therapy, irreversible electroporation, RFA, SBRT, and BT, were considered to improve postoperative preservation of sexual and urinary function compared with radical therapies. However, the lack of RCTs and consistent follow-up led to the limited use of these modalities as the first-line treatment (139). Most recently, a meta-analysis quantitatively, including 150 studies, compared the efficacy and toxicity of RP, HIFU, cryotherapy, SBRT, LDR, and HDR for radio recurrent prostate cancer. Compared to RP (53%), they reveal the adjusted 5-year RFS for cryotherapy (57%, p = 0.4), HIFU (46%, p = 0.2), SBRT (56%, p = 0.8), HDR (58%, p = 0.2), and LDR (53%, p = 0.9). No significant differences were observed among these modalities. In terms of toxicity, the adjusted incidence of severe GU toxicity for SBRT (5.6%, p < 0.001), HDR (9.6%, p = 0.002), and LDR (9.6%, p = 0.001) were all significantly lower than RP (21%), while no differences were found in cryotherapy and HIFU. Besides, HDR yielded significantly lower severe GI toxicity than RP (0.0% *vs.* 1.5%, p = 0.003), without other differences among these therapies regarding severe GI toxicity (140).

Additionally, the risk of secondary malignancy after BT is also of concern, especially in patients with prostate cancer, which has a long-term survival. Zelefsky et al. (141) reported the different incidences of in-field and out-of-field secondary malignancies among the patients who were treated with intensity-modulated radiation therapy (IMRT) and BT (15% *vs.* 10%). However, a retrospective study in prostate cancer (n = 1,310), comparing the incidence of second malignancy in 1,310 cases with prostate cancer who underwent EBRT and I-125 BT, combined with the 2000 census date from National Cancer Institute’s Surveillance, Epidemiology, and End Results data set, demonstrated that the 10-year overall incidence of second malignancies was significantly higher in EBRT group compared to I-125 BT group (25% *vs.* 15%, p = 0.02). The significantly increased incidence of skin cancer in the EBRT group (10.6% *vs.* 3.3%, p = 0.004) compared to the I-125 BT group might account for the higher overall incidence in the EBRT group. No significant differences in SM incidence of all in-field cancers (bladder and rectal) were seen among the two cohorts (142). When comparing LDR-BT with HDR-BT, Murray et al. (143) found that the risk was lower for bladder cancer with LDR-BT and similar for rectal cancer with both techniques. In addition, seed migration after I-125 implantation did not increase the risk of second malignancies for prostate cancer patients. However, a longer follow-up was required to further investigate the correlation between seed migration with secondary malignancies (144).

Indeed, several factors constrain the study on secondary malignancies after prostate BT and EBRT, including long follow-up time, difficulty to detect a difference in institutional databases, lack of comparator cohort, and shortage of details regarding radiotherapy modalities in large database studies (145).

Furthermore, new findings took place in the field of LDR-BT for prostate cancer in the past few years, including new treatment modalities, new techniques, advances in treatment planning, and postoperative evaluations. Guimond et al. (146) reported that I-125 BT boost to the dominant intraprostatic lesion (defined by sextant biopsy) might improve the biochemical without increasing toxicity with regard to 7-year bDFS rate (96% *vs.* 89%, p = 0.188). A phase I randomized controlled trial conducted in I-125 LDR-BT for prostate cancer demonstrated that a machine learning-based prostate implant planning algorithm had the potential to produce more favorable postoperative dosimetry and operational efficiencies compared with the conventional treatment planner (147). Postoperative dosimetry evaluation by CT or MRI is the important quality assessment step of I-125 BT. A prospective study for prostate cancer confirmed that fully balanced steady-state free precession and advanced MRI scan technique could acquire resultant image superior to the current clinical standard without using endorectal coil, which brought better patient tolerance, lower costs, higher clinical throughput, and higher precision (148). PSA is deemed an important indicator of biochemical control for posttreatment prostate cancer. There is not yet a definitive value of PSA to define the biochemical cure. Recently, through the comprehensive analysis of the association between different PSA ranges and DFS under different treatment modalities, a multicenter study identified PSA <0.2 ng/ml at 4 years after LDR prostate BT as a threshold value to predict the long-term (10–15 years) freedom from prostate cancer (149). Nevertheless, how long is the “long-term”? Lazarev et al. (150) reported their study, which identified 757 men with localized prostate cancer who underwent definitive LDR-BT, suggesting that 17-year rates for biochemical failure-free survival (BFFS) (79%), DMFS (97%), prostate cancer specific survival (PCaSS) (97%), and OS (72%). They also revealed the 17-year BFFS (86%, 80%, and 65%, p < 0.001) and OS (82%, 73%, and 60%, p = 0.09) rate for low-, intermediate-, and high-risk patients (150). Another study also reported the 10-year actuarial freedom from biochemical failure (FFbF) (52.0%), PCaSS (77.8%), and OS (56.7%) in patients who underwent salvage LDR-BT for biopsy-confirmed intra-prostatic recurrence after EBRT (151). Placement of hydrogel space between prostate and rectum immediately after I-125 implantation for prostate cancer significantly reduced RV150 and RV100, without impact on prostate V100 and V150, which had the potential to reduce late GI toxicity and improve bowel function (152). A novel optical fiber sensor was developed to monitor the real-time radiation during the BT, which was considered to guarantee the safety and efficacy of the I-125 BT procedure (153).

### Sarcoma

When it comes to sarcoma, a study enrolling 93 patients with metastatic soft tissue sarcoma after first-line chemotherapy failure suggested that I-125 BT significantly increases the PFS time (7.1 *vs.* 3.6 months, p < 0.001) with better symptom relief compared with second-line chemotherapy (81). A 12-year study, enrolling patients (n = 25) with locally recurrent head and neck soft tissue sarcoma after surgery and EBRT, reported the efficacy and safety of I-125 BT as a salvage strategy concerning objective response rate (76.0%), local PFS (median time 16.0 months), and OS (median time 28.0 months), with a few tolerable side effects (154). Of note, a cancer center has attempted to distinguish the difference in efficacy between I-125 BT and MWA on recurrent retroperitoneal liposarcomas (155).

### Others

I-125 BT has also achieved great success in the treatment of various other tumors. For recurrent cervical cancer after EBRT, I-125 BT is considered a reliable salvage treatment after initial EBRT and can mitigate cancer-related pain. However, factors including the recurrent site, tumor volume, and the prescribed dose impact the efficacy, as this strategy has been apt to cause pelvic wall recurrence (156, 157). In application to non-palpable ductal carcinoma *in situ*, I-125 can be utilized more effectively than wire-guided localization for guiding breast conservation surgery (158). Approximately 25% of patients with breast cancer are diagnosed with occult or non-palpable breast cancer (159). However, for these patients, the difficulty to define a clear margin for this type of lesion conflicts with breast conservation (160). Hence, I-125 BT preoperative localization as part of image-guided surgery was used to clarify the target margins and direct the breast conservation. Additionally, Chan et al. (161) reviewed the importance of I-125 BT in guided surgical excision of non-palpable breast cancer.

## I-125 Seed Stents in Hollow Organs

Primarily, I-125 has been used in the treatment of solid tumors. For a long time, the symptoms accompanying malignant obstruction in hollow organs decreased patients’ quality of life. For instance, concerning unresectable esophageal cancer, clinicians were primarily perplexed with how to relieve dysphagia and prolong survival. While SEMS relieved obstructions temporarily in esophageal cancer treatment, stent restenosis was a subsequent issue. Encouraged by the long-term benefits brought by intraluminal BT, which can deliver an effective localized high radiation dose to tumors elegantly, novel stents loaded with I-125 were developed (13). A preclinical study performed on normal pigs indicated that the specially designed I-125 seed stent inserted in the pancreatic duct was feasible and safe in an animal model, which provided experimental support for its use in the clinical practice of pancreatic cancer as well as in other scenarios (162).

In the 2000s, physicians attempted to evaluate the efficacy of I-125 loaded in a SEMS for inoperable esophageal cancer with dysphagia. The trial (n = 53) showed that the I-125 seed stent significantly improved the survival time (mean 8.3 *vs.* 3.5 months, p < 0.001) and delayed the time to stent restenosis (median 4.5 *vs.* 2 months, p < 0.044) (13). Because of this study, a multicenter, single-blind, randomized, phase 3 trial (n = 148) was conducted in China, further confirming the clinical efficacy of this type of irradiation stent on unresectable esophageal cancer, with prolonged survival (median 177 *vs.* 147 days, p = 0.0046) and a lower dysphagia score in the I-125 seed stent treatment group. Additionally, the same team also further investigated which factors can predict the OS outcome and relieve dysphagia with the I-125 seed stent (11).

Inspired by the successful utility of I-125 seed stent in esophageal cancer, two key trials (n = 23 and n = 328) were conducted to assess the efficacy in malignant biliary obstruction (12, 15). The latter trial, a multicenter, open-label, randomized, phase III trial (n = 328) comparing the irradiation stent with the uncovered SEMS found both stents were equally effective in relieving jaundice, but the I-125 seed stent was associated with significantly longer survival (median 202 days *vs.* 140 days; p = 0.020) and lower stent restenosis rate (21% *vs.* 33% at 360 days; p = 0.010) (12). Furthermore, a novel model was developed, on the basis of radiomics, *via* a retrospective study including 106 patients treated with an I-125 seed stent for unresectable pancreatic cancer combined with malignant biliary obstruction and predicted that the patients with slow progression are apt to have a longer restenosis-free survival (163).

Similarly, a prospective RCT was developed to evaluate the efficacy and safety of I-125 seed stents compared with conventional stents in 66 patients with unresectable malignant airway obstructions. Statistically and clinically significant improvement in OS (170 days *vs.* 123 days, p = 0.015) and a decrease in in-stent restenosis rate (21.2% *vs.* 45.45%, p = 0.037) were observed with the novel I-125 seed stent, without increased complications (14).

Although the I-125 seed-loaded stent has been introduced into clinical practice, they are not appropriate for all clinical scenarios, and the long-term toxicity and efficacy of this approach are still being determined for other tumors and lesions. The location of the lesion requiring treatment and its proximity to critical normal tissues with high sensitivity to radiation must be carefully considered.

## Biological Effects of Continuous Low-Dose-Rate Irradiation by I-125

Despite a large number of studies reporting the biological effects of EBRT (21), little attention is paid to I-125 BT, which generates continuous LDR irradiation. In the past decade, emerging evidence reported the radiobiological and immunological effects mediated by I-125. Of note, in contrast to EBRT, the continuous LDR irradiation could surmount problematic tumor growth that might occur during the EBRT interval, based on cell reoxygenation, cell cycle redistribution, cell repopulation, and cell repair. Concerning the radiobiological effects, it has been found that I-125 could inhibit cell proliferation *in vitro* and delay tumor growth in tumor models by upregulating apoptosis- and cell arrest-related genes, such as BNIP3 and WNT9A, through irradiation-induced DNA methylation (164). Moreover, the signaling pathways participating in the apoptosis induced by I-125 were explored to include the downregulation of hypoxia-inducible factor (HIF)1α, vascular endothelial growth factor (VEGF), and matrix metalloproteinase (MMP)-2/MMP-9 (165, 166). Besides apoptosis, I-125 seed radiation was found to activate the phosphoinositide 3-kinase (PI3K)/AKT signaling pathway to induce paraptosis-like cell death and trigger mitophagy by elevating mitochondria reactive oxygen species (ROS) level *via* the HIF1α-BCL2/Adenovirus E1B 19kDa Interacting Protein 3 (BNIP3)-NIP-3-Like Protein X (NIX) signaling pathway (167, 168). Given that mitophagy is an autoregulatory cell mechanism, it might be an option for potential targeted therapy to enhance the efficacy of I-125 BT when used in tandem. In addition, the Warburg effect is suppressed by I-125 seed radiation *via* upregulating miR-338 to inhibit 6-phosphofructokinase (PFKL), as the Warburg effect is deemed to be the landmark metabolic process of malignant tumors (169).

Additionally, the efforts to improve the clinical performance of I-125 for use in LDR-BT continue. A preclinical study performed on HCC models showed that the induced apoptosis and tumoricidal effect by I-125 seed radiation were facilitated by lobaplatin through the upregulation of the protein kinase RNA-like ER kinase (PERK)-eukaryotic initiation factor 2α (eIF2a)-Activating Transcription Factor 4 (ATF4)-CCAAT Enhancer Binding Protein homologous protein (CHOP) pathway (170). Moreover, hampering the expression of PERK can counteract the synergistic antitumor effect of lobaplatin on I-125 seed radiation, which predicated the potential combination therapy in future clinical practice (170). A novel cancer-targeting agent, known as folic acid-conjugated selenium nanoparticles, was developed to augment the I-125-mediated tumoricidal effects through increasing ROS and activating the mitogen-activated protein kinase (MAPK) and P53 pathways, resulting in DNA damage (171). Furthermore, the expression of DNA-PKCs, which are regarded as the key factors for DNA repair in the non-homologous end-joining pathway, was found to predict biochemical recurrence in prostate cancer after I-125 BT (172).

Theoretically, localized continuous radiation is assumed to yield an immune response analogous to EBRT. One study concerning the immune status after I-125 BT indicated that the frequency of CD3^+^T cells, CD4^+^T cells, and CD3^-^CD16^+^/CD56^+^NK cells increased significantly after the treatment, with a decrease of blood tumor markers (173, 174).

Difficulties in immunological studies of I-125 seed radiation include the construction of irradiated animal models, relatively lengthy observation periods, and the required dose to induce a local and/or systemic immune response. More preclinical and clinical investigations are needed to further investigate the immunomodulation of I-125 seed radiation.

## Perspective

### High-Level Randomized Clinical Trials

RCTs are vital to inform medical decisions and improve cancer care, and all patients who wish to undergo I-125 BT should be encouraged to take part in RCTs. Currently, the majority of clinical trials focusing on I-125 BT were single-armed or single-center. This has led to preliminary data showing I-125 BT as a safe and effective alternative to EBRT or surgery with a low risk of complications for patients with recurrent or inoperable lesions. The limitations of sample size and lack of control have restricted the ability to obtain high-level evidence-based conclusions for use in clinical practice and the optimization of guidelines. In addition, the benefit of I-125 BT in many tumors remains to be evaluated, given the lack of evidence of greater tumor control or less toxicity with this approach. Many in the oncology community are eagerly awaiting data from RCTs that compare current radiotherapeutic techniques with I-125 BT. Therefore, multicenter double-blind RCTs should be conducted to confirm the clinical efficacy in terms of I-125 BT.

### The Introduction of Artificial Intelligence Navigation With I-125 BT

The combination of AI and robotic technology has spawned advances such as cyber-knife, which has played a critical role in the modern use of SBRT. Therefore, it might be not only feasible but also beneficial to introduce AI navigator systems into I-125 BT. A clinical trial has been carried out on AI navigator-guided I-125 BT in some cancer centers to assess whether it can facilitate the accuracy of I-125 seed localization and shorter operation time. Thus, the integration of multiple advanced technologies would promote the popularization of I-125 BT.

### Investigation of Immunological Effects I-125 BT

The low-dose rate continuous irradiation-related biological effects are complicated. The underlying mechanism of I-125-induced apoptosis and tumor-killing effects remains fully unrevealed. Besides, the changes of the local tumor immune microenvironment (TIME) and the followed alteration of the systemic immune state after I-125 BT are still elusive. The question remains: Is the immunomodulation by I-125 analogous to EBRT, such as enhanced immune recognition and local accumulation of immunosuppressive factors? The prospect of exploring the optimal regimen of immunotherapy with I-125 BT is promising. Future investigation should help to determine the immunological effects of I-125.

### I-125 BT in Combination With Other Strategies

As the preclinical studies suggested, the third-generation platinum drug, lobaplatin, can enhance the tumoricidal effects of I-125. This may indicate that combinations of I-125 seeds with chemotherapy agents might be of interest in the future. Moreover, the autoprotective mechanisms of tumor cells, such as mitophagy, provide a potential therapeutic target (167, 168). Future experimental studies on the radioimmunological effects of I-125 may elucidate multiple therapeutic strategies based on the systemic immunomodulation of I-125.

## Conclusion

A rapid advance of technology has progressively increased the accuracy of I-125 BT, reduced unexpected adverse effects, and substantially improved survival. Consequently, the use of I-125 BT has expanded to multiple sites and the dose of radiation has been escalated with this technique. This has given rise to improvements in disease control. I-125 BT not only provides an opportunity to preserve organs that otherwise would have been surgically removed but also plays a critical role in the salvage of recurrent and refractory cancer. The endeavor to increase the accuracy, improve the LC, and minimize the side effects of I-125 BT continues to evolve.

## Author Contributions

SW collected all materials and wrote the manuscript. CL assisted with material collection and article writing and reviewed the manuscript. ML, YX, YJ, HS and BQ provided valuable comments for the manuscript. JW and CL conceived the core idea of the study, designed the article framework, and supervised this study. All authors contributed to the article and approved the submitted version.

## Funding

This work was supported by the National Natural Science Foundation of China (82073335 to JW and 81803051 to CL), the Natural Science Foundation of Beijing Municipality (7192220 to CL), and the China Postdoctoral Science Foundation (2018T110015 to CL), the China Postdoctoral Science Foundation (2017M620545 to CL).

## Conflict of Interest

The authors declare that the research was conducted in the absence of any commercial or financial relationships that could be construed as a potential conflict of interest.

## Publisher’s Note

All claims expressed in this article are solely those of the authors and do not necessarily represent those of their affiliated organizations, or those of the publisher, the editors and the reviewers. Any product that may be evaluated in this article, or claim that may be made by its manufacturer, is not guaranteed or endorsed by the publisher.
